# Clustering HLA Class I Superfamilies Using Structural Interaction Patterns

**DOI:** 10.1371/journal.pone.0086655

**Published:** 2014-01-27

**Authors:** Sumitro Harjanto, Lisa F. P. Ng, Joo Chuan Tong

**Affiliations:** 1 Duke-National University of Singapore Graduate Medical School, Singapore, Singapore; 2 Department of Biochemistry, Yong Loo Lin School of Medicine, National University of Singapore, Singapore, Singapore; 3 Singapore Immunology Network, Agency for Science, Technology and Research, Singapore, Singapore; 4 Institute of High Performance Computing, Agency for Science, Technology and Research, Singapore, Singapore; University of Queensland, Australia

## Abstract

Human leukocyte antigen (HLA) class I molecules are critical components of the cell-mediated immune system that bind and present intracellular antigenic peptides to CD8^+^ T cell receptors. To understand the interaction mechanism underlying human leukocyte antigen (HLA) class I specificity in detail, we studied the structural interaction characteristics of 16,393 nonameric peptides binding to 58 HLA-A and -B molecules. Our analysis showed for the first time that HLA-peptide intermolecular bonding patterns vary among different alleles and may be grouped in a superfamily dependent manner. Through the use of these HLA class I ‘fingerprints’, a high resolution HLA class I superfamily classification schema was developed. This classification is capable of separating HLA alleles into well resolved, non-overlapping clusters, which is consistent with known HLA superfamily definitions. Such structural interaction approach serves as an excellent alternative to the traditional methods of HLA superfamily definitions that use peptide binding motifs or receptor information, and will help identify appropriate antigens suitable for broad-based subunit vaccine design.

## Introduction

Human leukocyte antigen (HLA) class I molecules are cell surface glycoproteins that play a critical role in cell-mediated immune response [1]. They bind peptides derived from intracellular pathogens and present them to CD8^+^ T cell receptors [2]. T cell recognition of ligated HLA complex will initiate a cascade of immunological events that leads to the clearance of pathogens. The HLA binding site contains polymorphic cavities (or ‘pockets’) that fit the side-chains of complementary (i.e. anchor) residues on the binding peptide [3], [4]. It is known that specific HLA alleles can bind peptides with similar anchor residues and lead to the definition of “peptide motif” for an array of class I and II alleles [5], [6]. The subsequent discovery that certain HLA alleles can recognize very similar motifs resulted in the definition of HLA “supermotifs” or “supertypes” [7].

The characterization and classification of HLA alleles into superfamilies is important for the development of epitope-based vaccines [8]–[11]. By clustering HLA alleles on the basis of their structural features and/or peptide binding specificities, promiscuous T cell epitopes that bind multiple HLA alleles can be identified. Such peptides are key targets for the design of broad-based vaccines and immunotherapeutics because they are applicable to higher proportions of human population. However, experimental determination of binding specificities for even a single HLA allele is an expensive, laborious and time consuming process; and not practical for the study of HLA superfamilies that involve large numbers of alleles [12]–[14]. *In silico*, bioinformatics has been emerging as an alternative and viable approach for the classification of HLA superfamilies [15]–[22]. A number of clustering methods for HLA superfamily definitions are available, including those based on local sequence similarities in binding pockets [15]–[17], global sequence similarities [18]–[19] and peptide binding motifs [20]. Where data is limited or there is bias in the experimental binding motifs, mixed results have been reported [23]. Previously, Doytchinova and colleagues [14], [24] employed the use of hierarchical clustering and principal component analysis to classify HLA alleles according to their primary sequences and structures. The approach successfully identified HLA class I and class II supertype fingerprints and illustrated that only 1–3 amino acids are sufficient for an allele to be classified within a particular supertype. Kangueane *et al*. [25] defined critical polymorphic functional residue positions within the binding grooves of HLA-A, -B and -C alleles and grouped 47% of 295 HLA-A alleles, 44% of 540 HLA-B alleles and 35% of 156 HLA-C alleles to 36, 71 and 18 groups, respectively.

In this study, we explored the use of intermolecular bonding patterns for in-depth analysis of 58 HLA-A and -B binding characteristics. Our analysis showed that peptide/HLA structural interaction patterns vary among different alleles and may be grouped in a superfamily dependent manner. The results obtained here shed new light into HLA superfamily definition, further suggesting that HLA superfamily definitions may not be limited to peptide binding motifs or receptor information. Instead, they can be characterized at the intermolecular level that is based on the interactions between HLA proteins and their associated peptides, and consistent with solutions from X-ray crystallography. Through the use of structural interaction parameters described herein, a novel HLA class I superfamily classification schema has been developed for alleles with available binding sequences.

## Results and Discussion

### Significant Interactions

A total of 317 HLA-peptide interactions were identified using the homology models of 16,393 HLA-peptide complexes. Out of these, 230 interactions have less than 5% standard deviation in their supports across all alleles. All of these interactions, with the exception of H(159,1), have very low average supports below 6.92%. These interactions do not serve well to differentiate the binding specificities of the HLA alleles and were not used for further analysis. The remaining 87 HLA-peptide interactions, with more than 5% standard deviation in interaction support, were extracted and used as feature vectors representing the interaction profiles of the alleles.

**Figure 1:**
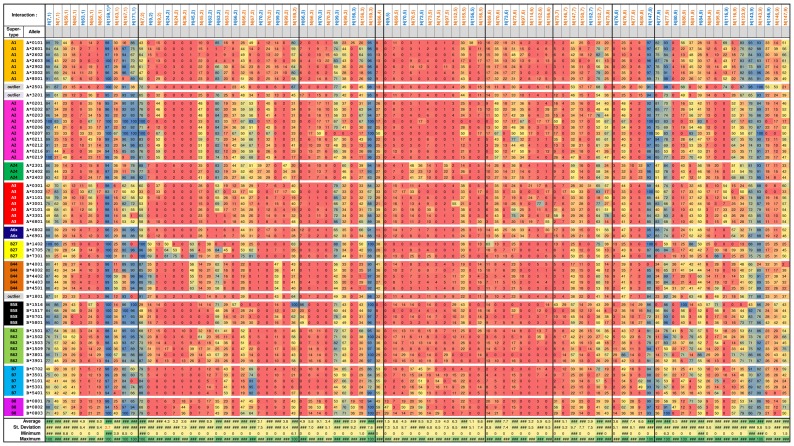
Heatmap of HLA-peptide interaction supports. HLA-A and -B alleles are sorted according to their assigned superfamilies and then by their allelic name. HLA-peptide interactions are sorted according to the interacting position on peptide ligand and then the position on the HLA molecule. The 88 columns consist of the 87 interactions with more than 5% standard deviation in their supports and H(159,1), which has exceptionally high supports (average of 97.5%) with low standard deviation (2.74%). Each interaction support value is color coded from red (0% support) to blue (100% support). The interactions are sorted from left to right; first by peptide position then by HLA position involved in the interaction.

As shown in Figure 1, most of the significant interactions (with more than 50% supports) are associated to the first three and last positions of nonameric peptides, which is consistent with existing HLA binding motifs [26], [27]. H(159,1) and N(159,3) exhibited very high average supports of 97.5% and 91.9% across all alleles. Tyr_159_ is conserved across all HLA-A and -B alleles associated with the current study and it has been observed to interact with all 20 naturally occurring amino acids on the amino terminal of the peptide (position 1). This lack of selectivity in hydrogen bonding suggests that the binding is independent of peptide side chain. The exact atoms found to be involved in H(159,1) are the carboxyl oxygen on the peptide backbone and the hydroxyl hydrogen on the side chain of Tyr_159_. H(147,8) and N(143,9) have also been found to have high average supports of 88.3% and 86.0% for all the alleles except B*4001. For B*4001, amino acid substitutions of Trp_143_ and Thr_147_ with Leu_143_ and Ser_147_ resulted in a complete loss of hydrogen bonding at position 8 of the peptide, and weakened hydrophobic interactions (24.1% support) at position 9 of the peptide.

The majority of the HLA-peptide interactions exhibited differences in their interaction supports in a superfamily-dependent manner. For example, the supports for H(7,1) are lower in A3 and B7 alleles with an average of 49.2% and 55.6% respectively, compared to the average support of 86.0% in the rest of the alleles. Such superfamily-dependent variability is more predominant in interactions involving positions 1, 2 and 9 of the nonameric peptides, whereas interactions involving the remainder positions are mostly uniform across the alleles.

### HLA-A Superfamilies

Five main clusters were observed: A1 (A*0101, A*2601, A*2602, A*2603, A*2902, A*3002, A*8001), A2 (A*0201, A*0202, A*0203, A*0205, A*0206, A*0207, A*0211, A*0212, A*0216, A*0219), A3 (A*0301, A*0302, A*1101, A*3001, A*3101, A*3301, A*6801), A24 (A*2301, A*2402, A*2403), and A6X (A*6802, A*6901). Hydrophobic interactions between peptide position 6 and receptor positions 70 and 73 are predominantly found in HLA-A alleles, compared to their HLA-B counterparts.

**Figure 2:**
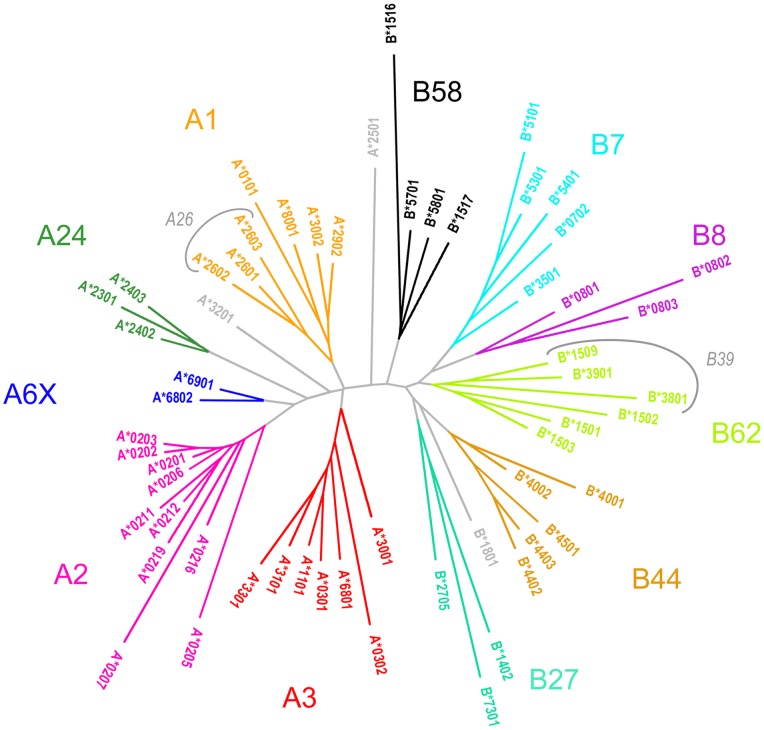
Dendogram based on Manhattan pair-wise distance matrix. A total of 11 clusters, 5-A and 6 HLA-B clusters, are derived from the clade topology and color-coded accordingly. The outliers (A*2501, A*3201, B*1801) are shown as single-leaf branches in light grey color. A26 and B39 superfamilies, which are defined by Lund *et al*. [20], manifest as sub-clusters under A1 and B62 respectively and are demarcated by grey-colored arches.

A1 alleles generally have very similar interaction profiles compared to A3 alleles. This is consistent with the proximity of the two clusters as shown in Figure 2. Although >20% differences in average support between the two superfamilies can be observed in H(7,1), N(63,1), H(171,1), N(159,2), N(66,4) and N(123,9), there are no interactions which are exclusive to either A1 or A3 superfamilies. N(123,9) appears unique to A1 alleles (∼79.0% support). Comparable supports in N(123,9) are not observed in all other superfamilies except B8 (∼67.4%), which is intriguing given that Tyr_123_ is conserved in all the alleles in this study.

**Figure 3:**
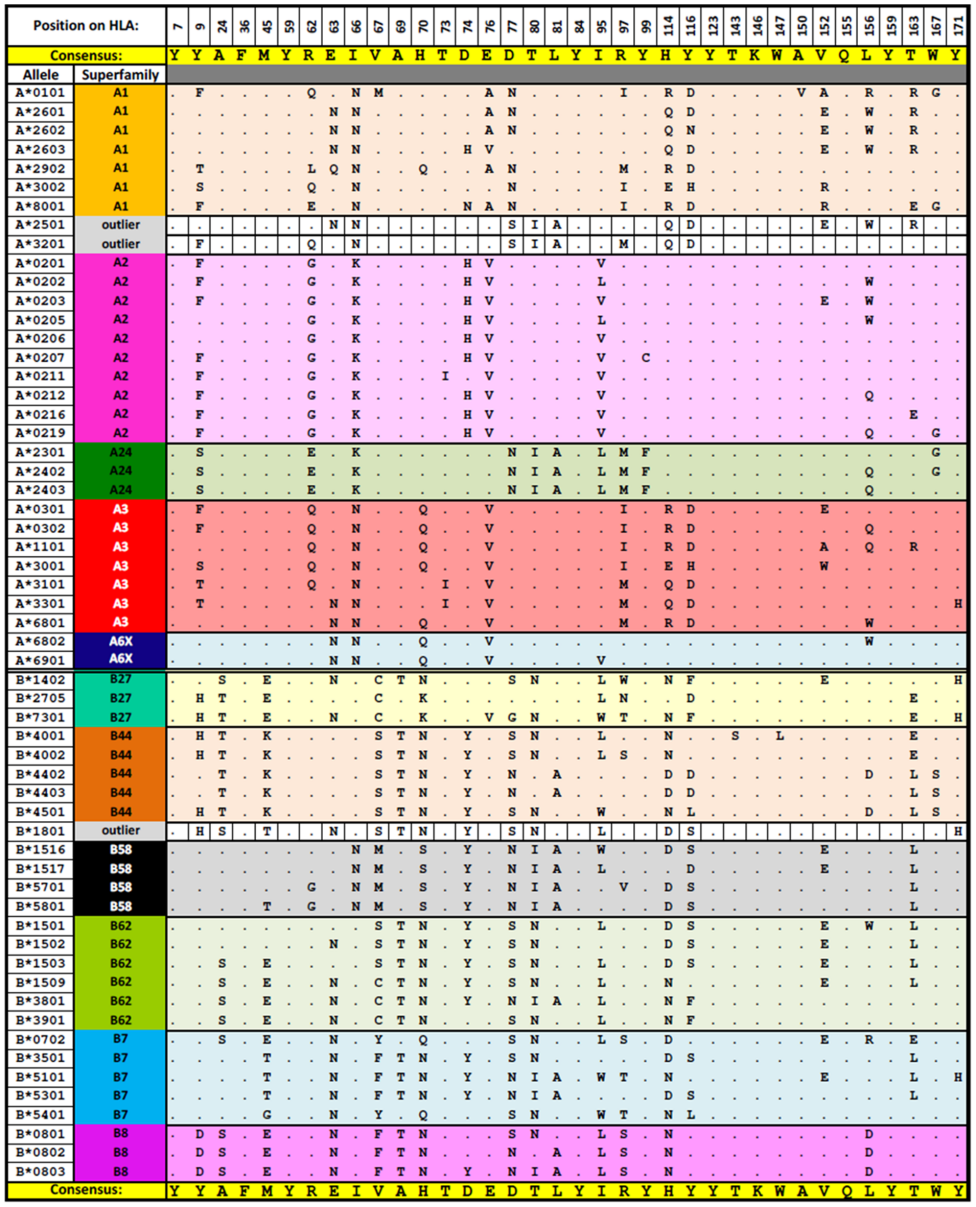
Amino acid residues that occupy the 36 critical positions on the HLA-A and -B molecules. The 36 positions are involved in the 88 interactions shown in Figure 1. The consensus residue, which occurs with highest frequency, in each position is shown on the second and the last row in the table in yellow shading. ‘.’ (a dot) represents that the allele possesses the same amino acid as the consensus residue.

A2 alleles, on the other hand, have interaction profiles similar to those in A24 and A6X. At position 2 of the peptide, comparable levels of support are observed in A2 and A24 alleles for majority of the interactions, except for N(9,2), H(70,2) and N(99,2). For A2 alleles except A*0205/06, hydrophobic interactions between peptide position 2 and Phe_9_ appears to be a dominant trait of the superfamily, and its substitution with Ser in A24 alleles resulted in the complete loss of hydrophobic interaction at this specific position. At peptide position 9, H(77,9) and N(81,9) have higher supports in A2 compared to A24 alleles. Similar loss of hydrophobic interaction is observed for all the A24 alleles whenever Leu is substituted with Ala at position 81 of the HLA molecule (Figure 3). A loss of hydrogen bonding is also observed when the predominant Tyr_99_ is replaced by Phe_99_ in A24 alleles. A24 alleles also seem to interact with peptide position 5 more frequently as compared to the rest of the alleles in the other HLA-A superfamilies; as observed with the relatively higher supports in N(70,5), H(73,5) and N(97,5).

As shown in Figure 3, A3 binding peptide repertoire is characterized by a strong preference for positively charged basic residues in position 9. This could be attributed to the combination of acidic residues: Asp_77_ and Glu_114/_Asp_116_, which is unique to the A3 alleles. The A*3201 allele, which lies in close proximity to the A3 alleles in the dendrogram (Figure 2), however, does not exhibit the same preference for basic residues on peptide position 9 as A3 alleles do, and neither does the allele possess the combination of acidic residues mentioned above. The Asp_77_ conserved among A3 alleles is replaced by the neutral Ser_77_ residue in A*3201. Therefore, it is likely that A*3201 does not belong to the A3 superfamily as proposed by Doytchinova *et al*. [23].

In previous classifications [20], [24], [28], [29], A*6802 and A*6901 (herein referred to as A6X) have been grouped under the A2 superfamily. The sequences of A2 and A6X are highly conserved in the F pocket region, which interacts with the C terminal region of the peptides. Nine out of 10 positions in this pocket are fully conserved (77, 80, 81, 84, 116, 123, 143, 146, and 147), while position 95 is occupied by either Val, Leu or Ile. The two superfamilies lie close to each other within the dendrogram (Figure 2) and exhibited highly similar support levels for all interactions involving peptide position 9. However, clear differences between the interaction profiles of these two superfamilies could be observed at peptide positions 1 and 2. N(63,1), N(7,2), N(9,2), N(45,2), H(63,2), H(66,2), N(67,2), and N(70,2) have significantly lower supports (>20%) in A6X compared to A2 alleles. Furthermore, the hydrophobic interaction N(62,1), which is completely absent in all A2 alleles, has a support of 18.7% in A*6802 and 14.0% in A*6901. Hence, we have classified A*6802 and A*6901 as a separate superfamily.

### HLA-B Superfamilies

Six clusters were obtained, with B*1801 excluded as an outlier: B7 (B*0702, B*3501, B*5101, B*5301, B*5401), B8 (B*0801, B*0802, B*0803), B27 (B*1402, B*2705, B*7301), B44 (B*4001, B*4002, B*4402, B*4403, B*4501), B58 (B*1516, B*1517, B*5701, B*5801), and B62 (B*1501, B*1502, B*1503, B*1509, B*3801, B*3901). In general, hydrogen bonding interaction H(45,2) and H(62,2) are exclusive to B27, B44, B*1509, B*3801, and B*3901, and are missing in the HLA-A alleles. Similarly, H(9,2) is also more highly supported (∼34.6%) in B27, B44, B58, B62 superfamilies and B*1801 as compared to HLA-A alleles (∼2.6% support).

B7 alleles have few significant interactions observed in the B-pocket region, which interacts with peptide position 2. A characteristic of this superfamily is high support level (∼84.6%) for hydrophobic interaction between Tyr/Phe_67_ of B7 alleles and peptide position 2.

The N(36,2) hydrophobic interaction is a distinctive characteristic of B8 (18.4–44.1% support) and B62 (≤12.9% support) alleles. The serologically defined B8 alleles have been classified as outliers by Sette and Sidney [30], and Sidney *et al*. [28]. In general, the binding peptide repertoire of B8 is distinctively different from the other superfamilies. The B8 alleles were shown to exhibit preference for hydrophobic residues on peptide position 9, and basic residues Lys and Arg at the third and fifth positions. H(156,3) hydrogen bond is another interaction unique to the B8 alleles. It is formed between the acidic Asp_156_ residue, which is conserved across the B8 alleles and basic residues at peptide position 3.

Among the HLA-B superfamilies, B27 alleles have the most diverse interaction profiles (Figure 1). This is likely a manifestation of the effect of small numbers of binding peptides collected for B*1402 and B*7301; with 8 and 16 peptides respectively. The loss of H(171,1) hydrogen bond as observed in B*1402 and B*7301, is directly linked to the substitution of Tyr_171_ by His_171_. This association is also observed in A*3301, B*1801 and B*5101.

Strong preference (97.4%) for negatively charged residues at peptide position 2 is observed in B44 alleles. This preference is attributable to the presence of the positively charged Lys_45_, which is unique to B44 alleles. Similar observation is made with the B27 and B39 alleles, where the negatively charged Glu_45_ is prevalent at the B-pocket.

Similar to B44 alleles, a strong preference (74.4%) for acidic residues at peptide position 2, and hydrogen bonding at H(99,2) is observed for B*1801, which has been classified under B44 and B7 superfamilies by earlier methods [24], [28], [29]. However, H(45,2) and N(45,2), which occur with average supports of 55.6% and 15.3% in B44 alleles, are entirely absent in B*1801, which contains the polar uncharged Thr_45_ instead of the positively charged Lys_45_ in B44 alleles. Thr_45_ is also found in some of the B7 alleles (B*3501, B*5101, B*5301) and H(45,2) and N(45,2) are also completely absent in these B7 alleles. While the binding repertoire of B*1801 clearly resembles that of the B44 alleles and Thr_45_ is found both in B*1801 and some B7 alleles, the interaction profile of B*1801 does not conform fully to either of the two superfamilies.

Compared to the other alleles, relatively high supports (∼47.4%) of H(66,3) are observed exclusively for B58 alleles among the HLA-B family. While some HLA-A alleles also possess Asn_66_, the support for H(66,3) is much lower (≤17.4%) among the HLA-A alleles.

### Superfamily Assignment

**Figure 4:**
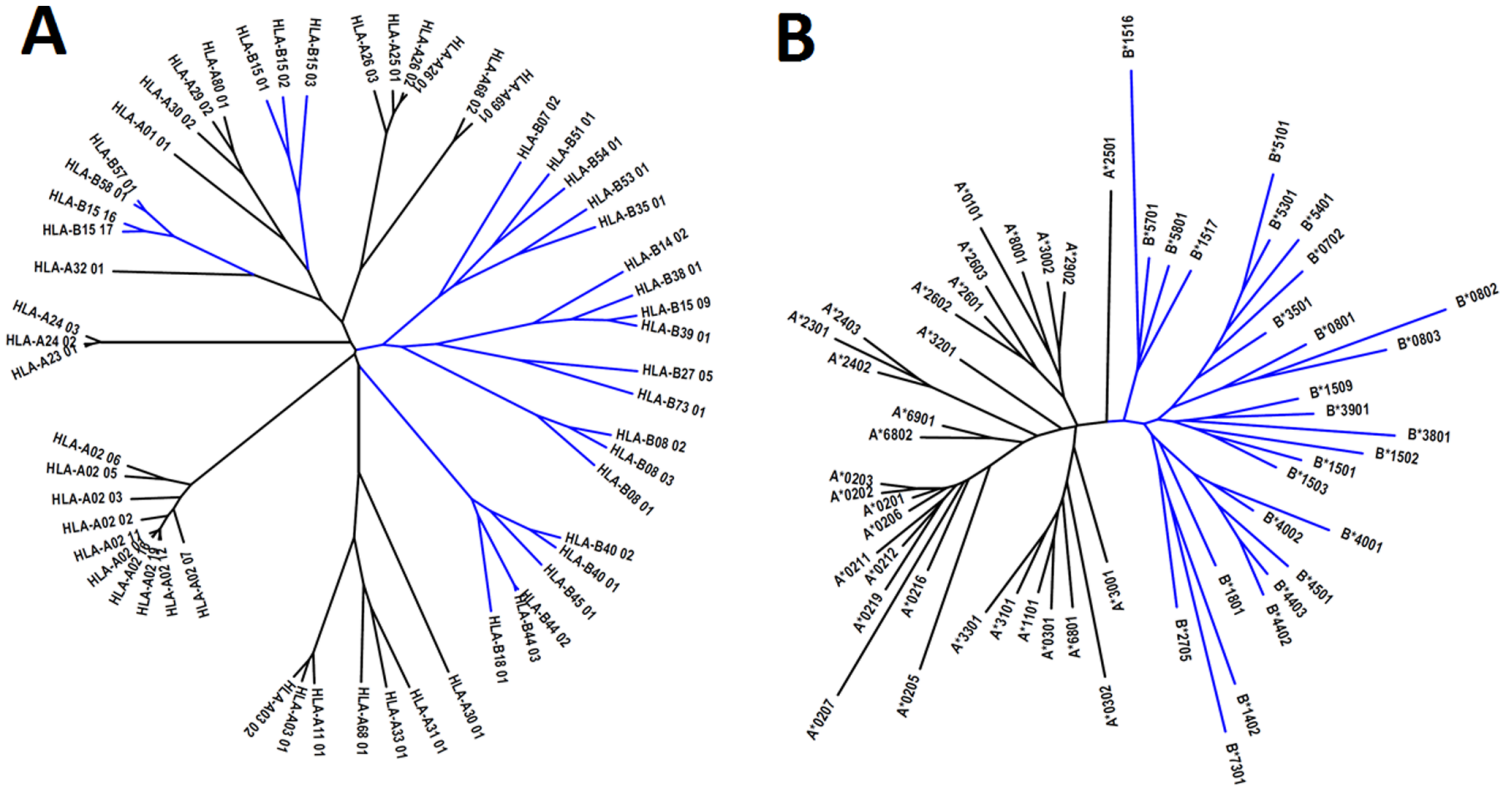
Dendrogram showing HLA-A and -B clusters generated using A) MHCcluster 2.0 Server [22], and B) our proposed method for the 58 alleles used in this study. Clear separation of HLA-A and -B alleles into well resolved, non-overlapping clusters could be obtained using our classification method, but not using the MHCcluster method. Blue: HLA-B alleles, black: HLA-A alleles.

The superfamily assignment to the alleles derived through our classification method (Table 1) is compared to previous studies [20], [24], [28], [29]. Previous efforts to cluster HLA class I alleles have consistently obtained cross-loci superfamilies comprising of alleles from more than one locus; in Lund’s attempt [20], A*2902 and B*1506 are grouped under the B44 and A1 superfamilies respectively; in Reche and Reinherz’s classification [20], apart from several new cross-loci superfamilies (namely ABX and B15) proposed, B*3801 is classified under the A24 superfamily; in Hertz and Yanover’s classification [29] based on learned peptide distance function, A*2902 is classified under the B44 superfamily and several alleles of B locus are grouped under A1 (B*1501, B*1516, B*1517, B*5701 and B*5702), A2 (B*1512, B*1513, and B*1518) and A24 (B*5801) superfamilies. Using our method, it is conceivable to separate the HLA-A and -B alleles into well-resolved, non-overlapping subtrees on the dendogram; with none of the HLA-A alleles classified under the HLA-B superfamilies and vice versa. A noteworthy observation is that some of these alleles (A*2902, B*1516, B*1517, B*5701, and B*5801) which were assigned cross-loci superfamilies in prior classifications could be found near the boundary demarcating the HLA-A and -B subtrees in our dendogram (Figure 2). Such observations clearly indicate that previous methods may not be precise, and potential wrong clustering could now be corrected using the new classification method described here (Figure 4). The consensus between the various methods and the one in this study, defined as the proportion of the common alleles assigned to the same superfamily in both methods compared, is given in Table 1. For A*2902 and A*3001, which are assigned dual superfamilies by Sidney *et al*. [28], they are now considered to be in agreement if they are assigned to either superfamily in this method shown here.

**Table 1:** Superfamily assignment.

HLA Allele	No. of Complex	This Study	Sidney *et al.* [28]	Hertz & Yanover [29]		Lund *et al.* [20]	Doytchinova *et al.* [24]	
				Peptide∧	BS*		COMSIA	MIF
**A** * **0101**	310	A1	A1	A1	A1	A1	A3	A3
**A** * **2601**	153	A1	A1	A1	*A26*	*A26*	A2	A3
**A** * **2602**	67	A1	A1	A1	*A26*	*A26*	A2	A3
**A** * **2603**	23	A1	A1	A2	*A26*	*A26*	A2	A3
**A** * **2902**	349	A1	A1 A24	B44	A1	outlier	A3	A3
**A** * **3002**	243	A1	A1	A1	A1	A1	A3	A3
**A** * **8001**	65	A1	A1	–	–	A1	A3	A3
**A** * **2501**	47	outlier	A1	–	–	A1	A2	A3
**A** * **3201**	310	outlier	A1	–	–	A1	A3	A3
**A** * **0201**	2775	A2	A2	A2	A2	A2	A2	A2
**A** * **0202**	752	A2	A2	A2	A2	A2	A2	A2
**A** * **0203**	785	A2	A2	A2	A2	A2	A2	A2
**A** * **0205**	6	A2	A2	A2	A2	A2	A2	A2
**A** * **0206**	694	A2	A2	A2	A2	A2	A2	A2
**A** * **0207**	6	A2	A2	A2	A2	A2	A2	A2
**A** * **0211**	88	A2	A2	–	–	–	A2	A2
**A** * **0212**	102	A2	A2	–	–	–	A2	A2
**A** * **0216**	34	A2	A2	–	–	–	A2	A2
**A** * **0219**	47	A2	A2	–	–	–	A2	A2
**A** * **2301**	192	A24	A24	–	–	A24	A24	A24
**A** * **2402**	822	A24	A24	A24	A24	A24	A24	A24
**A** * **2403**	59	A24	A24	–	–	A24	A24	A24
**A** * **0301**	865	A3	A3	A3	A1	A3	A3	A3
**A** * **0302**	6	A3	A3	–	–	–	A3	A3
**A** * **1101**	1161	A3	A3	A3	A1	A3	A3	A3
**A** * **3001**	438	A3	A1 A3	A1	A1	A1	A3	A3
**A** * **3101**	514	A3	A3	A3	–	A3	A3	A3
**A** * **3301**	215	A3	A3	A3	A3	A3	A3	A3
**A** * **6801**	578	A3	A3	A3	A3	A3	A3	A3
**A** * **6802**	497	A6X	A2	A2	A2	A2	A2	A2
**A** * **6901**	86	A6X	A2	A2	A2	A2	A2	A2
**B** * **1402**	8	B27	B27	–	–	outlier	B7	B7
**B** * **2705**	76	B27	B27	B27	–	B27	B27	B27
**B** * **7301**	16	B27	B27	B27	B27	outlier	B7	B7
**B** * **4001**	133	B44	B44	B7	B44	B44	B27	outlier
**B** * **4002**	162	B44	B44	B44	B44	B44	B27	outlier
**B** * **4402**	91	B44	B44	B44	B44	B44	B44	B44
**B** * **4403**	91	B44	B44	B44	B44	B44	B44	B44
**B** * **4501**	97	B44	B44	–	–	B44	B27	B7
**B** * **1801**	90	outlier	B44	B44	B7	–	B7	B7
**B** * **1516**	7	B58	B58	A1	B58	outlier	B44	B44
**B** * **1517**	25	B58	B58	A1	B58	outlier	B44	B44
**B** * **5701**	29	B58	B58	A1	B58	B58	B44	B44
**B** * **5801**	128	B58	B58	A24	B58	B58	B44	B44
**B** * **1501**	782	B62	B62	A1	B62	B62	B27	B7
**B** * **1502**	66	B62	B62	B62	B62	B62	B7	B7
**B** * **1503**	349	B62	B27	B62	B27	B62	B27	B7
**B** * **1509**	36	B62	B27	*B39*	*B39*	*B39*	B7	B7
**B** * **3801**	7	B62	B27	*B39*	*B39*	*B39*	B44	B27
**B** * **3901**	31	B62	B27	*B39*	*B39*	*B39*	B7	outlier
**B** * **0702**	470	B7	B7	B7	B7	B7	B7	B7
**B** * **3501**	393	B7	B7	B7	B7	B7	B7	B7
**B** * **5101**	165	B7	B7	B7	B7	B7	B44	B44
**B** * **5301**	176	B7	B7	B7	B7	B7	B44	B44
**B** * **5401**	129	B7	B7	B7	B7	B7	B7	B7
**B** * **0801**	499	B8	B8	–	–	outlier	B7	B7
**B** * **0802**	34	B8	B8	–	–	B8	B44	B44
**B** * **0803**	14	B8	B8	–	–	–	B7	B7

Comparison of our results against earlier classifications by Sidney *et al.* [28], Hertz and Yanover’s methods [29]; which include both peptide and binding site approaches; Lund *et al.* [20], and both methods by Doytchinova *et al.* [24]; which are based on COMSIA (Comparative Similarity Index Analysis) and MIF (Molecular Interaction Fields).

‘-’ denotes that the allele is not included for classification in the particular study.

* Superfamily definition based on learned distance function over the binding site of the alleles.

∧ Superfamily definition based on peptide-peptide learned distance function.

The average agreement in the classification of HLA-A alleles is 75.3%. For this locus, our results and that of Sidney *et al*. [28] is the highest (93.1%) among all other methods compared; the only two HLA-A alleles which are not in agreement with Sidney *et al*. [28] classification are A*6801 and A*6802, which are assigned to a new superfamily A6X. Our superfamily assignment to A2 and A24 alleles are in perfect agreement with all prior assignment by other classifications (Table 1). For A1 alleles, the disagreement arises from assignment of A26 superfamily proposed by Lund *et al*. [20] and Hertz and Yanover’s [29] binding site approach. However, it is interesting to observe that the A26 alleles defined by Lund *et al*. [20] and Hertz and Yanover’s [29], to cluster as a subtree within the A1 clade in the dendogram. Some disagreements are observed for A3 superfamily, where A*0301 was assigned A1 superfamily by Lund *et al*. [20] and A*3001, A*0301 and A*1101 by Hertz and Yanover [29] using the protein-based approach.

The average percentage agreement for the HLA-B alleles is 59.3%. This is mainly due to a low consensus (23.1 and 26.1%) with the classification of Doytchinova *et al.* [24]. This is due to the fact that B58, B62 and B8 superfamilies found in all the other studies, were not defined in the work of Doytchinova *et al*. [24]. For this locus, our results found high agreements with that of Sidney *et al.* [28] (84.6%), Lund *et al.* [20] (85.0%) and Hertz and Yanover’s [29] binding site approach (80.0%). However, a lower percentage agreement of 57.1% is observed between our result and Hertz and Yanover’s [29] peptide-based approach. This could be caused by disagreements observed for alleles clustered under B58 (0%) and B62 (33.3%) superfamilies. All the alleles in B58 superfamily are classified by Hertz and Yanover’s [29] peptide-based approach as under HLA-A superfamilies (A1 and A24), and three out of the six alleles in our B62 superfamily are assigned to the B39, which is defined only in Lund *et al*. [20] and Hertz and Yanover’s work [29]. Generally, the low consensus observed for B62 alleles (25%) is either due to the absence of the superfamily definition in the work of Doytchinova *et al.* [21] or the assignment of B*1509, B*3801 and B*3901 to the B27 and B39 superfamilies in the other methods.

Using HLA-peptide interaction patterns, we showed for the first time that HLA-A and -B alleles could be grouped in a superfamily dependent manner that is consistent with known HLA superfamily definitions. This method would not only serve as an alternative to the traditional binding motif-based approach, but could also separate HLA alleles at a higher specificity than current state-of-art. The use of generalized interaction profiles instead of HLA binding motifs would address current limitations in clustering the less-studied HLA molecules, and path the way for the grouping of HLA molecules with poorly characterized binding motifs.

## Methods

### Data

A total of 16,393 non-redundant nonameric binding peptide sequences from 58 HLA-A and -B alleles, which have a minimum of six binding peptides each, were retrieved from the Immune Epitope Database (IEDB) [31]. The sequences of the corresponding HLA class I alleles were extracted from the IMGT/HLA sequence database [32].

### Template Assignment

The crystallographic structures of 90 HLA class I peptide complexes from 17 HLA-A and -B alleles were extracted from the Protein Data Bank (PDB) [33] and used as templates for homology modeling. Template assignment was performed using a scoring function that incorporates both HLA and peptide homology to measure the suitability between each target sequence and the templates. Pair-wise sequence similarities, *S(C_1_,C_2_)*, between target and template HLA class I alleles, *C_1_* and *C_2_*, were estimated using the Henikoff/Tillier Probability Matrix from Blocks (PMB) [34] as implemented in the Protdist program from the PHYLIP software package [35], where 0≤ *S(C_1_,C_2_) ≤*1, with 0 and 1 denoting 0% and 100% similarity respectively. For a given peptide alignment, the degree of conservation at position *i*, *V(i)*, was defined as the difference between the maximum entropy and the observed entropy in that position, i.e. *V(i)* = log_2_ *N*−*(E(i)+e(n))*, where *N* ( = 20) is the total number of equi-probable amino acid types, *E(i)* = −∑_(*all x*)_ *P(x,i)* log_2_ *P(x,i)* is the observed entropy at position *i* where *x* is one of 20 amino acid types, and *e(n)* is a correction factor for datasets with few sample sequences [36]. *P(x,i),* the probability of occurrence of amino acid *x* in position *i*, is estimated by *F(x,i)*, the frequency of amino acid *x* at position *i* in the alignment. Thus, *P(x,i)*≈*F(x,i)* = *k(x,i)/L* where *k(x,i)* is the number of occurrence of amino acid *x* at position *i* and *L* is the height of the column in the alignment, which is equivalent to the number of sequences in the alignment. The scoring function *M* between two HLA-peptide complexes, *C_1_* and *C_2_*, is defined as *M(C_1_,C_2_) = S(C_1_,C_2_*)•∑(*V(i)•b_i_(C_1_,C_2_)*, where b_i_(C_1_,C_2_) is the BLOSUM62 substitution score [37] for amino acids at peptide position *i* of *C_1_* and *C_2_*. Thus, *M* measures the overall degree of conservation between the target and template ligands across all peptide positions, weighted by the observed conservation of amino acids among the templates at each position, and adjusted by the similarity between the template and target alleles. When the scores of two or more crystallographic structures are equal, the highest quality template with the best resolution was selected among the returned results. 571 HLA-peptide complexes which failed to obtain a positive *M* score were removed.

### Homology Modeling

The program MODELLER [38] was employed for comparative modeling of all 16,393 template assigned HLA-peptide complexes. The models were constructed by optimally satisfying spatial constraints obtained from the alignment of the template structure with the target sequence and from the CHARMM-22 force field [39].

### Intermolecular Hydrogen Bonds

The number of intermolecular hydrogen bonds between the bound peptide and MHC protein was calculated using HBPLUS [40] in which hydrogen bonds are defined in accordance to standard geometric parameters. Hydrogen bonding patterns of all complexes presented in this study are available in MPID-T [41] (http://surya.bic.nus.edu.sg/mpidt).

### HLA-peptide Interactions

Intermolecular interactions between the bound peptide and MHC protein were calculated using the program LIGPLOT [42] in which hydrogen bonds and hydrophobic contacts are defined in accordance to standard geometric parameters. We define H(*r*,*p*) and N(*r*,*p*) as hydrogen bonding and hydrophobic interactions, respectively, between position *r* on HLA molecule and position *p* on the peptide ligand. We further define the support of an interaction for an allele as the percentage of occurrence of the interaction across all the HLA-peptide complexes involving the allele. The average support of an interaction is its supports averaged across all alleles in this study.

### Clustering of HLA-peptide Interactions

A Manhattan pair-wise distance matrix was constructed to quantify the differences between the interaction profiles of each allelic pair. The Fitch-Margoliash algorithm [43] was then applied for clustering the alleles using the distance matrix. A total of 1,000 trees were generated by randomizing the input order of alleles, and optimization was performed through global rearrangement of subtrees in each iteration of tree construction. Finally, the tree with the lowest average percent standard deviation (APSD) was used in this study. Clusters were derived based on the topology of the clades observed in the unrooted dendrogram (Figure 3). The branch lengths of the tree are scaled to the inter-allele distances, as specified in the Manhattan pair-wise distance matrix with an APSD of 8.163%. The percent standard deviations observed, which represent estimates of the standard errors incurred by the inter-allele distances depicted on the dendogram, range from 0% to 14.194%. The alleles are color-coded according to the topology of the respective clades that define the clusters.
